# Reproductive success is predicted by social dynamics and kinship in managed animal populations

**DOI:** 10.12688/f1000research.8713.1

**Published:** 2016-05-11

**Authors:** Saul J. Newman, Simon Eyre, Catherine H. Kimble, Mauricio Arcos-Burgos, Carolyn Hogg, Simon Easteal

**Affiliations:** 1 John Curtin School of Medical Research, Australian National University, Acton, Australia; 2 Wellington Zoo, Wellington, New Zealand; 3 Sedgwick County Zoo, Wichita, USA; 4 Zoo and Aquarium Association Australasia, Sydney, Australia

**Keywords:** Conservation, social interaction, kin selection, meerkats, group dynamics

## Abstract

Kin and group interactions are important determinants of reproductive success in many species. Their optimization could, therefore, potentially improve the productivity and breeding success of managed populations used for agricultural and conservation purposes. Here we demonstrate this potential using a novel approach to measure and predict the effect of kin and group dynamics on reproductive output in a well-known species, the meerkat *Suricata suricatta*. Variation in social dynamics predicts 30% of the individual variation in reproductive success of this species in managed populations, and accurately forecasts reproductive output at least two years into the future. Optimization of social dynamics in captive meerkat populations doubles their projected reproductive output. These results demonstrate the utility of a quantitative approach to breeding programs informed by social and kinship dynamics. They suggest that this approach has great potential for improvements in the management of social endangered and agricultural species.

## Introduction

The growing global crisis in biodiversity has caused the survival of endangered species to become increasingly dependent on managed breeding programs. These are generally designed to maximize reproductive success ^1, 2^, but they sometimes fail to increase or even maintain population sizes ^1^. Similar pressures to increase reproductive success in managed social species exist in agriculture, where the maximization of reproductive output is often synonymous with increasing yield.

A possible reason for poor or underperforming outcomes is that kinship and group structures, which are known to affect breeding success in wild populations ^3– 6^, are suboptimal in managed populations. Therefore, it may be possible to improve the success of conservation and agricultural breeding programs involving social species by optimizing kin and group interactions.

Here we investigate this possibility using kin and group interactions inferred from detailed longitudinal records of kinship, migration between institutions and reproduction records maintained by zoological institutions. We apply novel methods to predict reproductive success in populations of the highly social meerkat, *Suricata suricatta*, from observed variation in age, sex, relatedness and social group structure.

Meerkats are an important model species in the development of evolutionary theory, providing an empirical test case for hypotheses about social evolution derived from both game and kin selection theory ^7– 9^. Game theory predicts that individuals will interact cooperatively or competitively to maximize payoffs in direct fitness ^10^, and that these interactions do not depend on kinship structure. In contrast, kin selection theory predicts that individuals will seek to maximize their inclusive fitness by modifying the direct fitness of biological kin ^11, 12^, implying that the fitness effects of social interactions between cohabiting individuals are significantly affected by kinship structure.

Testing the alternative predictions of these theories requires longitudinal life-history data on social dynamics, reproductive success, survival, and kinship structure that are difficult to obtain from wild populations, but which are available for some managed populations in zoos. Here we evaluate and compare the predictions of these two theoretical approaches with data for managed meerkat populations.

We obtained data for managed meerkat populations in North America and Australasia from zoological studbooks, which record extensive life history data about individual animals to a time resolution of one day across multiple decades. These genealogies were documented to avoid inbreeding, and records span more than 113 years, with comprehensive coverage across the last 50 years. Across this period there is complete documentation of location of > 99% of individuals in the population, the exact dates of their transfer between institutions, and of their birth and death dates.

Meerkat life histories can be accurately reconstructed on an individual level from these data. Interactions between managed meerkats occur in a finite, discrete geographic space: individuals either have the possibility of interacting within the same zoo in either breeding or non-breeding enclosures, or they are completely socially isolated from one another in separate enclosures. Coupled with detailed genealogical data, this property can be used to reconstruct detailed individual life histories of individual captive meerkats, including the number, sex and relatedness of all other individuals living in the same zoo.

**Figure 1.**
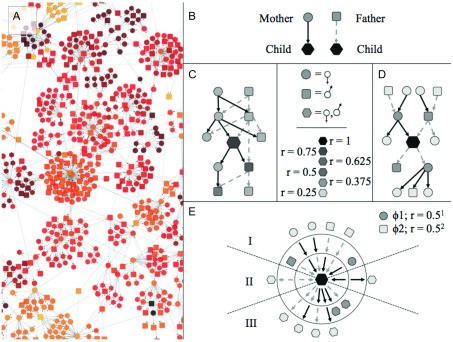
The standard inheritance graph or SIG. ( **A**) A section of the kinship-directed acyclic graph (kDAG) in Figure S1. ( **B**) Within the kDAG, nodes representing parents are connected to nodes representing their children by directed edges. ( **C**) Each individual in the graph is related to a finite sub-graph of individuals (coefficient of relatedness, *r*, indicated by shading in **C**– **E**). Even at small diameters, sub-graph structures can be highly variable (as between **C** and **D**). However all possible sub-graphs of a kDAG can be reduced to a finite set of paths with nodes grouped into ascendant (I), lateral (II) or descendant (III) classes ( **E**), without any loss of information on inheritance patterns or relatedness. This reduced set of paths is represented as a SIG ( Figure S5) in which nodes connected by ascendant paths (I) can include only one individual of each sex, and nodes in lateral (II) and descendant (III) SIG paths can contain any number of individuals of any sex.

**Figure S1.**
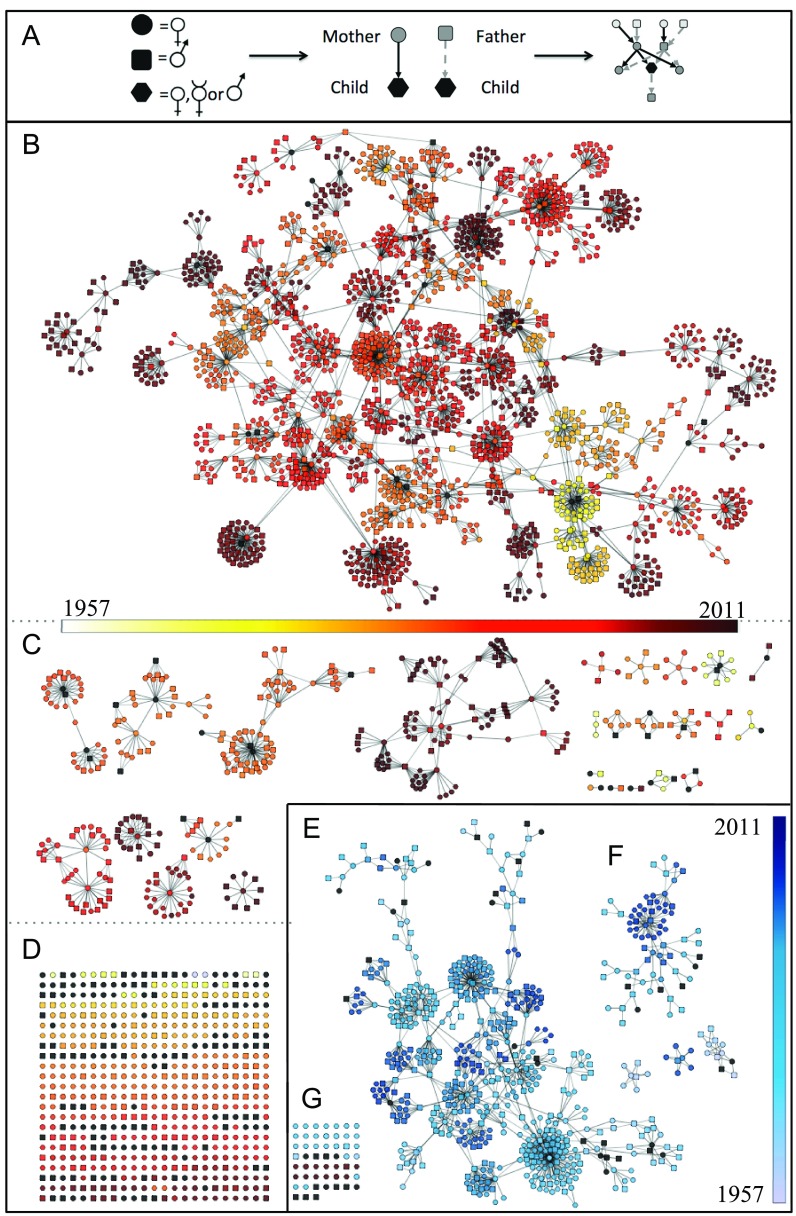
Genealogies represented as kinship directed acyclic graphs (kDAGs). Each individual is represented as a node and parent nodes are connected to offspring nodes by directed edges ( **A**), for the North American ( **B**– **D**) and Australasian ( **E**– **F**) meerkat populations. Both graphs collapse to one major cluster ( **B**, **E**), many smaller unconnected sub-graphs ( **C**, **F**), and a set of unconnected nodes ( **D**, **G**). Variation in birth dates is indicated by color gradients. Individuals with inaccurate (to month) or unknown dates of birth are shown as black nodes.

To quantify kinship in the complex and inbred multigenerational meerkat populations for which data are available, we initially mapped the relatedness of individuals in the populations as directed acyclic graphs of kinship (kDAGs), in which nodes represent individuals, and parents are connected to their children by directed edges ( Figure 1; Figure S1). We then evaluated standard approaches to estimating relatedness. All zoo-bred individuals with known parentage were included in our analysis.

## Materials and methods

Extensive demographic, geographic and genealogical data were obtained for two large independent managed meerkat populations, the North American Regional Meerkat Studbook ( 21; n = 2843) and the Australasian Regional Meerkat Studbook ( 22; n = 668). Within these populations, 2083 individuals (59%) have two documented parents and 973 (28%) have four documented grandparents. Of 1807 records of transfer between zoological institutions, 89% are accurate to the exact day, and less than 1 per cent of all individuals have been lost to follow-up. Our analysis was based only on records of meerkats living in zoos. We excluded records from other organizations such as animal suppliers and private owners, or institutions that did not have listed addresses (n = 23), which we recoded as ‘wild’. Entry of individual meerkats to zoos from these other institutions was treated as equivalent to entry from wild populations. No information about relationships among individuals at the data-collection baseline is available, and individuals entering the population (n = 185) were treated as unrelated. Individuals were also excluded from analysis if they had been lost to follow-up or released into the wild or wild-encoded institutions (n=237).

Birth, death and transfer dates were encoded numerically and rounded to the nearest month. We excluded dates that were not recorded to a time resolution of one month or for which uncertainty was indicated in the records (e.g., “1988” or “~Mar 1988”; ~13% of recorded dates).

The kinship structures of the managed meerkat populations were represented as kinship directed acyclic graphs (kDAGs; Figure S1) in which each node represents an individual connected to its offspring by a directed edge ( Figure S1A).

**Figure S2.**
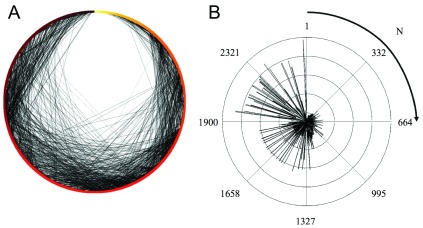
The growth in network complexity within the kDAG showing edge effects and node connectedness. By sorting the kDAG radially by order of entry into the population (date of entry indicated by color in **A**; order of entry (N) indicated by radial position in **A** and **B**), change in graph connectedness is indicated by the increasing density of directed edges over time ( **A**, grey edges) and the closeness centrality of nodes ( **B**). The closeness centrality of a node is a measure of the mean path length required to reach all nodes in a graph from that node ( *16*). Unlike other centrality measures, closeness centrality incorporates information from all paths between nodes, not just the shortest mean path. Higher closeness centrality indicates higher connectedness, with a baseline closeness centrality of zero for an unconnected node.

The extent and complexity of a meerkat’s kinship network changes over time and there is a general increase in the connectedness and complexity of the overall meerkat kDAG, as indicated by increased density of directed edges ( Figure S2A), and by the closeness centrality ^23^ of nodes ( Figure S2B). The addition of immigrant meerkats introduces variation in the rate and direction of these changes.

**Figure S3.**
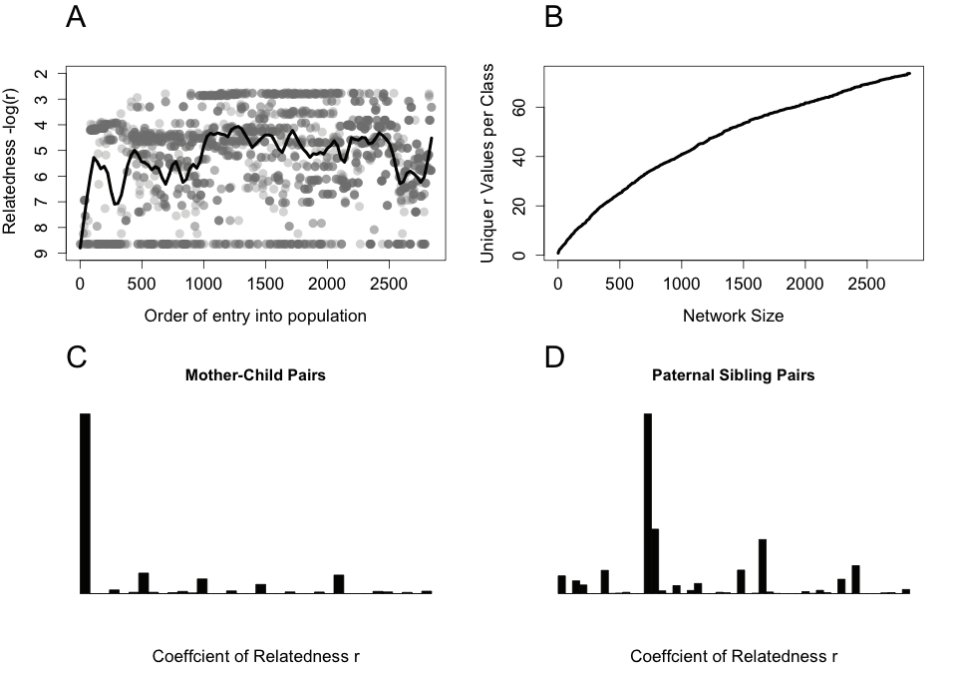
Effects of increasing network complexity on calculated coefficients of relatedness, *r*, in the meerkat population. ( **A**) The mean coefficient of relatedness of focal individuals to the rest of the population is dependent on the order in which they enter the genealogy (either through birth or immigration) and is driven by changes in network connectedness and complex edge effects. ( **B**) Individuals become increasingly connected by multiple relatedness paths as the diameter of the kinship network grows, increasing the number of unique values of *r* within each kinship category. ( **C**) For instance, *r* between mother-child pairs increase from a single value ( *r*=0.5) to 38 unique values as the kDAG increases in size. ( **D**) A similar increase in unique *r* values occurs between paternal sibling pairs. Traditional kinship categories do not capture this variation in relatedness coefficients within the pedigree.

The way genealogical information has been collected over time can bias results from traditional kinship measures. Estimating Wright’s coefficient of relatedness *r* ^13^ in our genealogy using the “kinship2” package ^24^ in the R environment version 3.1.2 ^25^, we found that the mean coefficient of relatedness between an individual and the general population ( *r _mean_*) was dependent on: order of entry into the genealogy, number of documented ancestors within the zoo system, and number of higher-degree relatives ( Figure S3).

Relatedness coefficients between kinship categories such as “mother” or “second cousin” are highly dependent on the degree of genealogical connectedness and completeness. Most mother-child pairs are related by exactly *r* = 0.5 when the genealogy is small. However, mother-child pairs may also be related by many other kinship categories, such as second and fourth cousins. As the number of individuals in the kDAG grows, higher-degree relationships became more completely documented, and an increasing number of mother-daughter pairs became connected by other relationship paths.

This accumulation of higher-degree relatedness continued until 39% of all mother-daughter pairs had coefficients of relatedness > 0.5 ( Figure S3C). As the kDAG size increases, an increasing number of individuals have a unique degree of inter-relatedness ( Figure S3B). The problem is more pronounced between individuals with primary connections through higher degree kinship categories such as “cousin” ( Figure S3D).

To overcome these biases we developed the standard inheritance graph (SIG) as a new method of quantifying kinship in these large genealogies.

Every individual within a non-hermaphroditic, exclusively sexual population has exactly two parents of opposite sex. The number of edge paths connecting any individual to their parents is fixed: every individual in a sexual population is connected to exactly one female parent by exactly one ascendant “maternal” edge, and one male parent by one ascendant “paternal” edge.

In a population of selfing hermaphrodites, all individuals in a population still have exactly one father and exactly one mother, but these “parents” may be the same organism. The number of parents is variable, but the number of paths connecting an individual to its ascendant kin is constant: individuals have exactly one ascendant maternal path and exactly one ascendant paternal path, which terminate at exactly one female and exactly one male, respectively. The set of paths connecting a focal individual to its first-degree ascendants is the same whether both parents are different individuals or the same individual.

**Figure S4.**
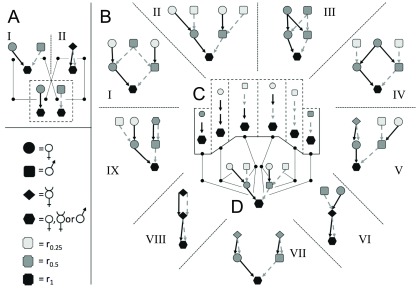
The existence of a universal set of ascendant paths in diverse genealogies. ( **A**) For any strictly 2-sex species, a genealogy of first-degree ascendants has only two possible states: an individual has either one mother and one father ( **A** I), or one selfing hermaphroditic parent ( **A** II). These two genealogies contain different numbers of individuals, related by different coefficients of relatedness, but they can be represented by identical set of “paths” linking parents of each sex to their offspring. In both cases a child has exactly one female parent and one male parent (as indicated within dashed-line box). The number of possible genealogies is therefore larger than the number of possible path combinations. ( **B**) This relationship holds at higher degrees of relatedness. At two degrees of relatedness, all 9 possible ascendant genealogies ( **B**; I–IX) possess a fixed set of exactly one each of the 6 paths shown in ( **C**). Each child has exactly one female parent, one male parent, one maternal and one paternal grandfather, and one maternal and one paternal grandmother, even if e.g., their maternal grandfather and their paternal grandfather is the same individual (as in **B** I). In this way, all genealogies of any given diameter have a single invariant set of ascendant paths, independent of their actual shape. ( **D**) This invariant set of paths can be represented as a directed acyclic graph in which each node represents a class of relative (e.g., maternal grandfather, paternal grandfather, etc.), rather than a distinct individual. An individual may occupy more than one node (as e.g., in **B** I, where the same individual is both maternal grandfather and paternal grandfather), but a node may not contain more than one individual in an ascendant lineage.

This invariant set of paths connecting a focal individual to a variable number of ascendants, exists at all higher degrees of relatedness ( Figure S4). An individual may have only two grandparents in an inbred lineage, but they still possess exactly four ascendant grandparental paths: a path of two ascendant male edges to their father’s father; a path of two ascendant female edges to their mother’s mother; a path of one ascendant female edge followed by one ascendant male edge to their mother’s father; and a path of one ascendant male edge followed by one ascendant edge to their father’s mother.

**Figure S5.**
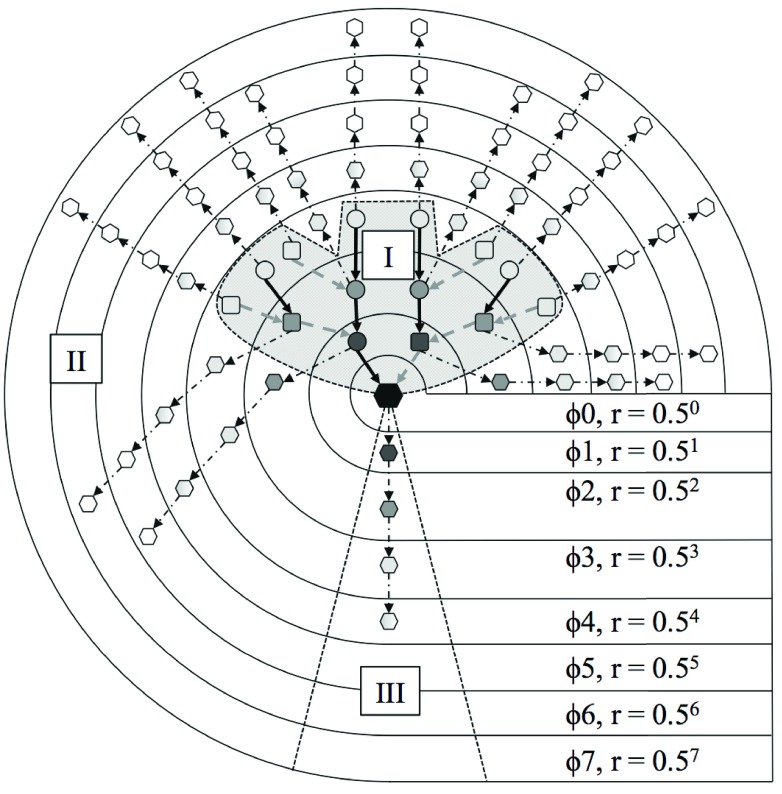
The use of SIGs in model testing. A 3-degree ascendant, 4-degree descendent SIG is shown with ascendant (I), descendant (III) and lateral (II) kin path sets identified as described in Figure 1. SIGs were constructed for all focal individuals (represented by the black node) who have a complete set of documented third-degree ascendant relatives (I), in which all their known relatives are represented. For each focal individual, the numbers of local and global kin (local kin and global kin variables in Table 1) in each ascendant (I) and lateral (II) kin class were counted each month. We estimated how well the reproductive success of focal ** individuals (descendent relatives in path set III) was predicted by cohabitation of kin classes in path sets I and II. ϕ = degree of relatedness.

This underlying structure extends to all genealogies with exactly two sexes. If all ascendant and non-ascendant paths are documented, in any genealogy of any size, the resultant set of paths falls into a single fixed set of possible paths ( Figure S4C, Figure S5). In addition, all ascendant relatives at φ degrees of relatedness are connected to a focal individual by exactly 2φ unique edge paths ( Figure S4B), and each ascendant path terminates at exactly one individual of a determinate sex.

The set of all ascendant and non-ascendant kinship informative paths has several useful qualities.

1). Each path makes a fixed contribution to the total coefficient of relatedness between two individuals. If an individual is connected to a relative by a path of length φ parent-child edges, this path contributes *k*φ to the total coefficient of relationship between these two individuals, where *k* is the coefficient of relatedness between a parent and child.

2). The coefficients of relatedness of different paths linking the same two individuals are additive. When and individual is connected to a relative by more than one path (e.g., when the same individual is both their maternal and paternal grandmother) the total coefficient of relatedness between them is equal to the sum of the coefficients of relatedness for all paths that connect them.

3). Every path is informative of structural relatedness, independent of variation in coefficients of relatedness. For instance, both a maternal and paternal ascendant path of length 1 terminates at an equally related relative, a ‘mother’ and ‘father’, who are related to the focal individual by *r* = k. However, these equally related relatives are structurally different classes in the ascendant path set.

Pedigrees typically reached at least one wild founder within 4 ascendant generations in the meerkat populations, limiting the number of higher degree relationship paths that could be reliably observed between individuals. In turn, this limited the diameter of completely documented SIGs below 4 degrees of relatedness. We therefore selected all ‘focal’ individuals in the managed populations with a full set of documented first, second or third degree ascendant relatives using a simple pattern-matching algorithm in R 3.1.2 ( 25; Supplementary material). We constructed 1, 2 or 3-degree ascendant SIGs for each of these focal individuals, using the direct descendants the focal individual and their ancestors.

Each focal individual’s reproductive success was estimated as the sum of relationship coefficients ( *r _III_*) to all direct descendent relatives over time (represented in the ‘III’ paths of the SIG in Figure 1F and Figure S5). For example, the reproductive success of a focal individual producing one child ( *r* = 0.5) and one grand-child ( *r* = 0.25) over the period *n…n+1* is:

$$\sum_{n}^{n+1}r_{III}=0.75,$$

By including multiple generations of descendent relatives, this metric accounts for differential reproductive success among immediate offspring, and avoids weighting the production of sterile and fecund offspring equally.

In our analysis, the number of predictor variables was high relative to the number of cases being predicted. Group size and kinship metrics were further constrained in that they included mixed binary (e.g., the presence/absence of mothers), discrete and continuous variables that did not generally have simple distributions. Predictive algorithms built using parametric approaches, such as logistic regression, perform poorly under these conditions ^26^.

We therefore constructed several recursively partitioned regression models (RPRMs) to predict reproductive success, using different sets of training variables. Built on a nonparametric learning algorithm, RPRMs can produce robust predictive models using large sets of variables of mixed data types. RPRMs are a robust data-driven technique of model construction ^26, 27^ that make no assumptions about the linearity of relationships and which are independent of variable ordering.

RPRMs begin by finding the value of a predictor variable that best partitions cases of the outcome variable (the ‘parent node’) into two smaller populations or ‘child nodes’. This ‘best’ partition is found by identifying all possible partitions of all variables, calculating the sum of squares between child nodes using a simple analysis of variance for each partition, and selecting the partition that leads to the maximum difference in the between-node sum of squares.

Each child node from this ‘best’ partition is then used as the parent node for a new round of partitioning. This splitting algorithm is applied recursively to all child nodes until one of three stopping criteria is satisfied. The splitting algorithm halts if a child node reaches a minimum sample size (the ‘minbucket’ parameter), a parent nodes contains too few cases to attempt a split (the ‘minsplit’ parameter), or when a split is unable to increase the fit of the model by a given factor (the ‘complexity parameter’).

In the absence of strict stopping criteria, this algorithm produces an over-fitted predictive model, represented by a binary decision tree of splitting criteria. We eliminated over-fitting in this model by pruning this decision tree using random-sampled cross validation.

To perform *k*-fold cross validation, the data were randomly subdivided into equally sized groups *G _1_…G _k_*. For each successive group *G _1_…G _k_*, the *ith* group *G _i_* was excluded and an RPRM was constructed from the remaining data. All sub-trees of this model were then used to predict the outcome variable of the excluded *G _i_* group. The accuracy of each sub-tree was measured as the *R ^2^* coefficient between predicted and observed values. After testing across all *k* groups, the sub-tree with the highest mean accuracy at predicting the outcome variable was selected out of all tested sub-trees. This selected binary tree represents the predictor most robust to variation introduced by sampling of the original data, and forms the output RPRM. Pruning RPRMs in this way effectively eliminates model over-fitting, returning a tree that robustly predicts the outcome variable ^26, 28^.

To further exclude the possibility of over-fitting, we externally cross-validated each RPRM in the Australasian meerkat data, data that was not used in model construction. The accuracy of each RPRM in predicting this external holdout population was calculated by measuring the correlation coefficient between the observed reproductive success in the Australasian meerkat population, and the reproductive success independently predicted by each RPRM in the North American population ( Table 1).

**Table 1.** Predictive accuracy ( *r* ^2^) of recursively partitioned regression models constructed for 1, 2 and 3 diameter SIGs.

		SIG diameter			
Model	Mean kinship size	1	2	3	Mean *r* ^2^
Local kin (α)	2.9	0.20	0.15	0.23	0.19
Non-local kin (β)	24.3	0.05	0.00	0.18	0.08
Global kin (α + β)	27.2	0.02	0.11	0.22	0.12
Pseudo kin (γ)	10.5	0.17	0.24	0.19	0.20
Sigma (α + γ)	13.4	-0.11	0.23	0.15	0.09
Split (α) & (γ)	13.4	0.17	0.31	0.39	0.29

RPRMs were constructed using the ‘rpart’ package version 4.1-1 in R 3.1.2 ^25^, using data from the North American meerkat population only ^21^. We used identical parameters for all RPRMs, splitting using the ‘anova’ method, with minsplit and minbucket values of n=15, and the default complexity parameter of 0.01. All models were pruned using internal 10-fold cross-validation.

All RPRMs were constructed using a base set of predictor variables, including individual age and sex, and the age-specific female, male and total group size within each zoo. Added to these predictor variables were each of the variables quantifying the number of real present relatives, the number of living absent relatives, the number of global relatives, and the number of pseudo-kin, for four initial predictive models.

All models were trained and tested using focal individuals with complete 1, 2 or 3-degree SIGs, producing twelve predictive models (‘local kin’, ‘non-local kin’, ‘global kin’ and ‘pseudo kin’ predictive models, across 1, 2 and 3-degree SIGs; Table 1).

Two subsequent models were constructed from the local kin and pseudo kin variables. The ‘Split’ model retained the distinction between kin and non-kin by treating pseudo kin and local kin as independent variables. The ‘sigma’ model was constructed using identical data, but eliminated the distinction between kin and non-kin by summing local kin and pseudo kin to form a new, combined kinship class.

All of these models were constructed using the North American meerkat population data and tested, using identical parameters, in the independent Australasian meerkat population.

We developed a combined genetic/packing algorithm that optimizes social group structure to increase the reproductive success of the meerkat populations under realistic constraints ( Figure S4). The aim was to predict how rearranging individuals and their relatives across zoos improved per capita reproductive success of the entire meerkat population.

The genetic/packing algorithm operated by generating 1000 seed populations by randomly assigning new locations, with replacement, to individuals. Seed populations were selected that did not exceed historic group sizes observed within a zoo over the past decade, and which did not reduce a zoo populations below thresholds shown in Table S1. These requirements formed simple packing constraints imposed in each randomization step of this algorithm.

A genetic optimization algorithm was applied to each seed population. Five “mutated” populations were generated for each seed population, by randomly sampling (with replacement) groups of 50 individuals from each seed population and randomly reassigning them to new locations. These five mutated populations were tested for adherence to the inbuilt group size (packing) constraints. If populations failed to satisfy these constraints, the mutation process was repeated until five randomized populations were obtained that could be accommodated within existing zoo enclosures.

The population-wide reproductive success of each of the five mutated populations (plus the original seed population) was then predicted under these new conditions using the ‘sigma’ predictive model. The algorithm selected the population rearrangement with the highest predicted reproductive success across a 12-month period under this model.

**Figure S6.**
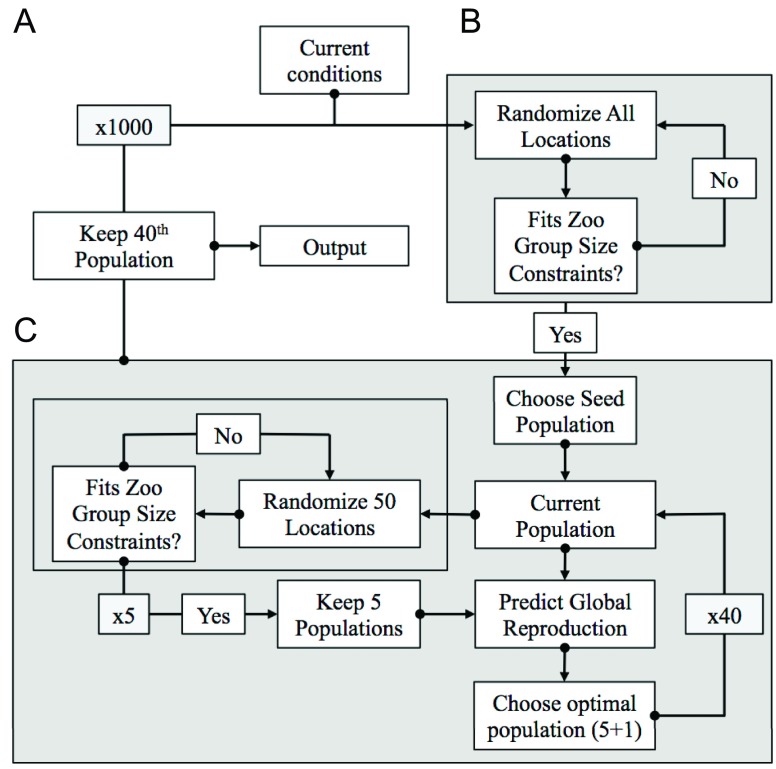
Map of the genetic/packing algorithm used to optimize the social group structure. ( **A**) We generated 1000 seed populations that satisfied group size constraints for all zoos, with individuals randomly assigned to locations. ( **B**) A local optimization algorithm was applied to each seed population. This algorithm generated “mutated” populations that randomly reassigned new locations to groups of 50 individuals from the seed population, rejecting populations that did not satisfy group size constraints, to generate five new populations. We forecast the overall reproductive success for these five “mutated” populations and the original population, and selected the global optimum of all six populations. ( **C**) The globally optimal population was then used to start the next cycle of mutation-selection, and the total process repeated 40 times.

This selected population was then used as the starting point for another identical round of mutation and selection. This entire process, of random population rearrangement within packing constraints, prediction of reproductive success under the new arrangement, and selection of the optimal population structure, was iterated 40 times for each of the initial 1000 seed populations ( Figure S6). We then selected the population structure with the highest predicted population-wide reproductive success over 12 months, under the ‘sigma’ model predictions ( Figure S6).

Same-sex siblings born into single paternity litters have perfectly congruent family trees. Furthermore, these individuals have identical ages, and often occupy the same enclosures: these ‘littermates’ therefore often share exactly identical kinship and social structures.

**Figure 2.**
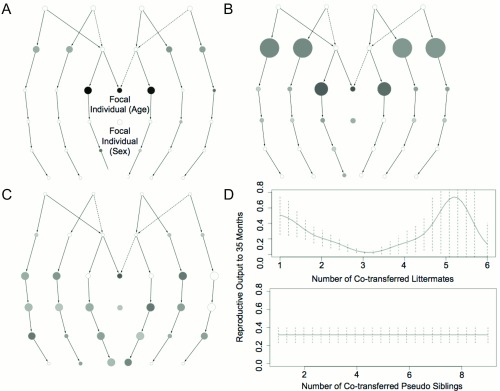
Effect of social cohabitation, age and sex on meerkat reproductive success. ( **A**– **C**) SIG classes are represented as a ‘crane-fly’ plot for local kin, non-local kin, and pseudo-kin models respectively, with number of individuals in each SIG class indicated by node diameter, and predictive effect indicated by gray scale. ( **D**) Observed changes in reproductive success after co-transferring same sex ‘littermates’ or unrelated ‘pseudo-siblings’ between institutions. Effect sizes are scaled independently in **A**– **C**.

We used this property of same-sex littermates to test the associations detected using the local kin and split models, where the best predictors of reproductive output were the numbers of cohabiting paternal and maternal siblings ( Figure 2A).

Individuals in both meerkat populations are routinely transferred between institutions, in groups of variable composition. We selected all individuals sent to new institutions within groups of more than one individual (a ‘co-transfer’ group). Individuals known to have reproduced before the date of first transfer were excluded to eliminate any bias against transferring highly reproductive individuals. We then compared the effect on reproductive output of co-transfer with littermates and co-transfer with unrelated individuals of similar age and sex using a Kruskal-Wallis rank sum test ^29^ in R version 3.1.2 ^25^.

Genetic measures of kinship, such as Wright’s coefficient of relatedness ^13^, were affected by the stochastic arrival of unrelated individuals into the kDAG over time; failed to distinguish between equally related but distinct classes of kin; and exhibited complex edge effects ( Figure S2, Figure S3). Relatedness coefficients also changed unpredictably over time in response to the changing connectedness and diameter of each kDAG, independent of actual changes in population structure ( Figure S3).

Kinship classes, such as “mother” or “second cousin” provide an alternative approach. However, these also failed to capture the complexity of kinship structures in the meerkat populations because individuals can belong to more than one kinship class. As a result, the total number of kinship classes and the number of unique combinations of classes connecting biological relatives increase nonlinearly with increasing kDAG diameter. Even in small genealogies, almost all individuals are related to one another by rare non-equivalent combinations of kinship classes ( Figure S3).

To overcome these limitations, we developed a new, unbiased approach to quantifying kinship based on ‘standard inheritance graphs’ (SIGs). This approach considerably reduced the complexity of genealogical networks while retaining all information on coefficients of relatedness and biological kinship structures.

We used the SIG approach to evaluate how kin- and non-kin-based social structure predicts reproductive success in the managed meerkat populations. We constructed a series of recursively partitioned regression models (RPRMs) to predict reproductive success from these variables, and the age and sex of the focal individual ^14^.

 For each month during the life of each focal individual, we constructed predictive models of reproductive success using several sets of predictor variables, using the number of: living relatives in each SIG class inhabiting the same zoo (local kin; α), living relatives in each SIG class inhabiting other zoos (non-local kin; β), living relatives in each SIG class, regardless of location (global kin; α + β), and unrelated individuals matching the age and sex of relatives in each SIG class that inhabited the same zoo (pseudo-kin; γ).

## Results

De-identified meerkat data and code README.txt contains a description of the contents. Instructions are included in the R code as comments. Click here for additional data file. Copyright: © 2016 Newman SJ et al. 2016 Data associated with the article are available under the terms of the Creative Commons Zero "No rights reserved" data waiver (CC0 1.0 Public domain dedication).

Variation in local kin predicted ~19% of the individual variation in reproductive output in the Australasian meerkat population ( Figure 2A, Table 1). Cohabitation with siblings and, to a lesser extent, uncles and aunts are the main contributors to this effect ( Figure 2A). There is some contribution from focal individual age but none from either group size or focal individual sex. The ~8% of variation predicted by non-local kin ( Table 1) follows a similar pattern ( Figure 2B). The similar pattern arises because, where the number of individuals in a kinship category is constrained ( *e.g.,* the ‘mother’ category, which always contains just one individual), the presence of local kin implies a reciprocal absence of non-local kin. A ‘local kin’ effect is, therefore, mirrored by a reciprocal ‘non-local kin’ effect.

The predictive value of pseudo kin (mean *r* ^2^ = 0.20) is similar to that of local kin. However, the two variables have distinct effects, as evident in the map of contributing model components ( Figure 2C). Furthermore, when the two variables are included as separate variables in the ‘split’ model, their combined predictive value (mean *r* ^2^ = 0.29) is substantially increased. In contrast, when kin and pseudo kin are combined as a single variable in the ‘sigma’ model their net predictive value (mean *r* ^2^ = 0.09) is substantially less than when these variables are treated individually, indicating that kin and non-kin have distinct non-additive effects that partially cancel each other out when kinship structure is ignored ( Table 1).

Patterns of animal transfers between zoos also indicate distinct kin and non-kin effects on reproductive success. Variation in the number of siblings co-transferred to a new zoo is a strong predictor ( *p* = 0.0002; Figure 2D) of reproductive success, with increased numbers of siblings associated with reduced reproductive output independent of litter size. In contrast, variation in the number of co-transferred non-siblings of the same sex and age does not predict subsequent reproductive success ( *p* = 0.39; Figure 2D).

Heritable variation in reproductive potential would result in a correlation between sibling number and subsequent reproductive success, which could confound these results. However, in these populations, when the effect of co-transfer is ignored, there is no correlation between sibling number and subsequent reproductive success ( *p* = 0.30). Furthermore, co-transfer with increasing numbers of siblings significantly reduced reproductive success across litters of the same initial size.

The observed local-kin and non-local kin effects could also be affected by inter-kin correlations caused by heritable variation in reproductive potential, if there was a tendency for members of families with low reproductive potential to be co-located, and those from families with high reproductive potential to be located in different enclosures. If this were the case, however, global kin would have better predictive accuracy than either local kin or non-local kin, which is not what we observe. Furthermore, heritable effects could not explain how different relative classes with equal coefficients of relatedness have different predictive effects on reproduction ( Figure 2A,B), or why the lifetime reproductive success of parents and offspring are not significantly correlated ( *p* = 0.59).

Thus, distinct kin and non-kin effects can be identified. When these effects are treated separately, approximately 30% of variation in reproductive success in managed meerkat populations can be predicted from current social conditions.

We extended this analysis to investigate whether previous reproductive success and current social conditions could accurately predict future reproductive success over a number of months. We used the split model to capture both kin and non-kin effects and included previous reproductive success as a predictor variable. The outcome variable predicted by these models was the sum of future reproductive success at 24 monthly intervals, excluding all cases where the focal individual died during this period.

**Figure 3.**
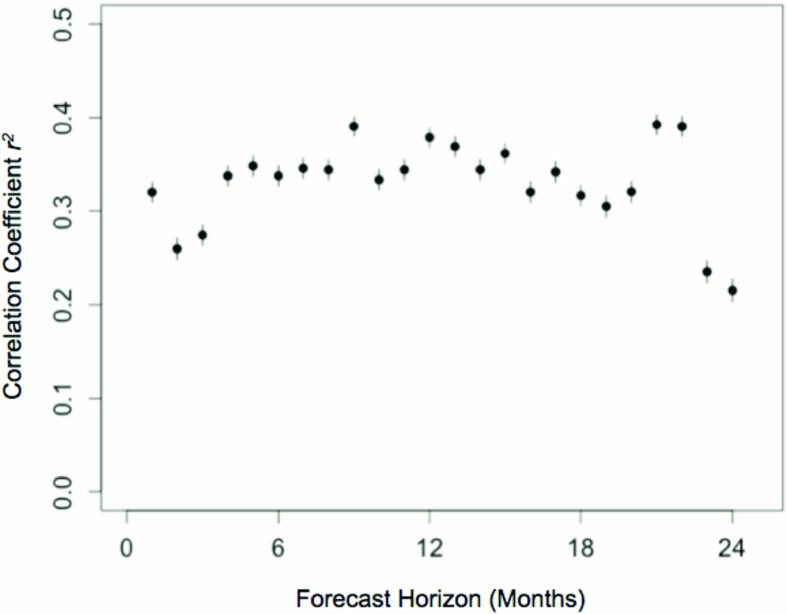
Prediction of future reproductive success in managed meerkat populations from current social conditions. Data on group and kinship structure in the North American meerkat population was used to train predictive models forecasting future reproductive success at monthly intervals up to 2 years. The predictive accuracy of these models was estimated as the correlation coefficient between predicted reproductive success based on the North American population and the reproductive success observed in the independent Australasian population ( *r* ^2^; 95% confidence intervals indicated by whiskers).

As before, RPRMs trained to predict future reproductive success in the North American population were tested in the independent Australasian population. The accuracy of these models, estimated as the correlation coefficient between predicted and observed values in the Australasian population, ranged from 0.21 to 0.40 over a two-year period ( Figure 3). Distinct local kin and pseudo kin effects were again evident in these models, in addition to the relatively strong effects of previous reproductive success and group size.

This result indicates that restructuring zoo populations to optimize their social composition could improve their future per capita reproductive success. We designed a recursive genetic optimization algorithm to explore this possibility further, taking account of the practical constraints of current zoo management practices ( Figure S6).

**Figure 4.**
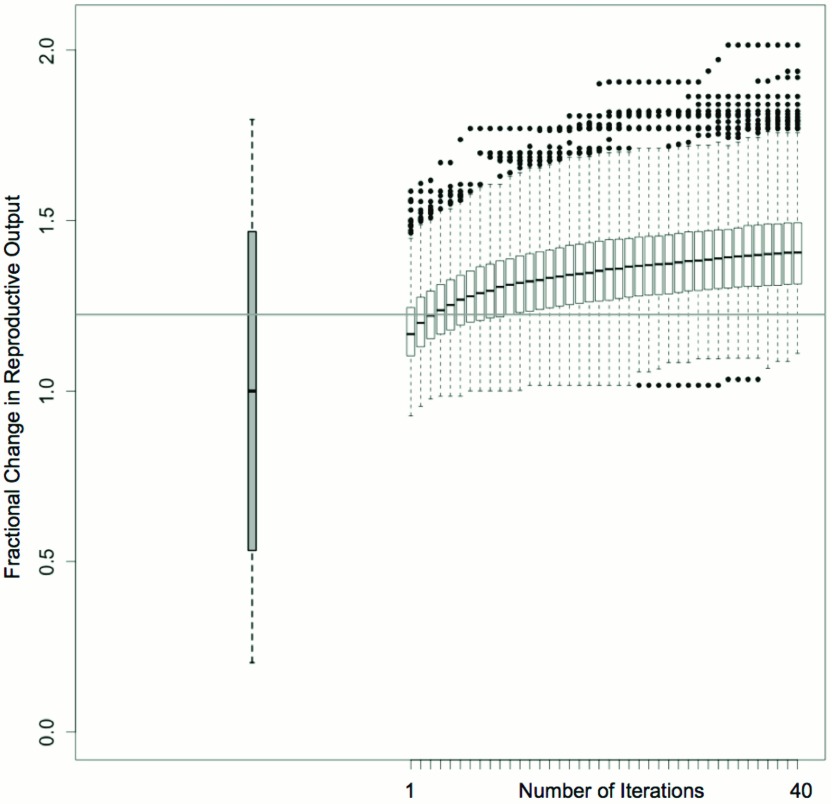
Predicted improvement of reproductive success from group structure optimization using a genetic algorithm. The resulting distribution of reproductive output scores are shown relative to the Holt-Winters (grey box) and recursively partitioned regression model (horizontal grey line) predictions for unaltered conditions ( Figure S6).

The algorithm was applied to 1000 randomly generated starting population rearrangements of the 521 living North American meerkats housed in 89 zoos ( Figure 4). Iterative optimization resulted in a general improvement in predicted 12-month reproductive output. The most optimal population configuration predicted a two-fold increase in the per capita reproductive success of the meerkat population compared to the baseline prediction ( Figure 4), an increase made possible by a high level of variation in offspring number between individuals and zoos.

## Discussion

These results demonstrate that both kin-kin cohabitation and general social structure have independent value in predicting reproductive success. Our analysis supports a non-exclusive role for both game and inclusive fitness theory interactions, reconciling findings from different species that support either strong kin selection ^4, 15^ or game-theoretic interactions ^10, 16^ as important determinants of reproductive success.

The predictive accuracy of our models was possible because we detected differential interactions between equally related but distinct classes of kin using the SIG method. This enabled finer segregation of kinship classes than can be achieved by existing methods.

This finding has clear implications for the management of social species. Our analysis suggests the considerable scope for improving the success of managed breeding programs by moving individuals into optimally or near-optimally structured social groups, which can be achieved within the constraints of existing enclosures. For instance, the observation of reduced reproduction in the presence of siblings is supported by data on incest avoidance in wild meerkat populations ^9^, and suggests a simple kinship-based mechanism for increasing reproductive success.

We developed this approach in the meerkat, a species of considerable scientific importance, but limited economic value. However, in principle the SIG approach is applicable to managed populations of any species of endangered or agricultural species exhibiting social behavior.

Most agricultural animals were domesticated to take advantage of their social herding behaviors ^17^, suggesting that there may be scope to improve their management through optimization of social or family structure. Some plant species display kin- and density-specific effects on survival, growth and productivity ^18– 20^, suggesting these traits might also be improved through optimization of kin structures in plants.

Kin-kin interactions within important agricultural species are often documented by large pedigrees from which high-degree kinship networks can be derived. We have focused here on reproductive success as an outcome, but this approach could be used to assess and modify the effect of social behavior on any trait from existing pedigree data, including agriculturally important traits such as growth rate and yield.

## Data and software availability

*F1000Research*: Dataset 1: De-identified meerkat data and code, 10.5256/f1000research.8713.d121675 ^30^

Due to concerns over animal-rights based opposition to managing social and endangered species, locations and names have been de-identified. Identified data is available on request from the Zoo and Aquarium Association Australasia or the American Zoo and Aquarium Association.

## Figures and Tables

**Table S1.** Group size reduction cutoffs used in the combined genetic/packing algorithm.

Current group size (# animals)	Acceptable group size reduction
1–5	0
6–8	1
9–12	3
13–15	4
16–19	5
20–25	8
25+	Any reduction down to current holdings
