# Supplementary material for: Reproductive success is predicted by social dynamics and kinship in managed animal populations
Source: F1000Res. 2016 May 11;5:870. [Version 1] doi: 10.12688/f1000research.8713.1 (PMC5155502; doi:10.12688/f1000research.8713.1)
Supplement: Supplementary file 2 [file f1000research-5-9375-s0001.tgz › 1ee9971b-acdd-4e4d-bec1-d8f23e985d20.docx]

**Supplementary material for ‘Reproductive success is predicted by social dynamics and kinship in managed animal populations’**


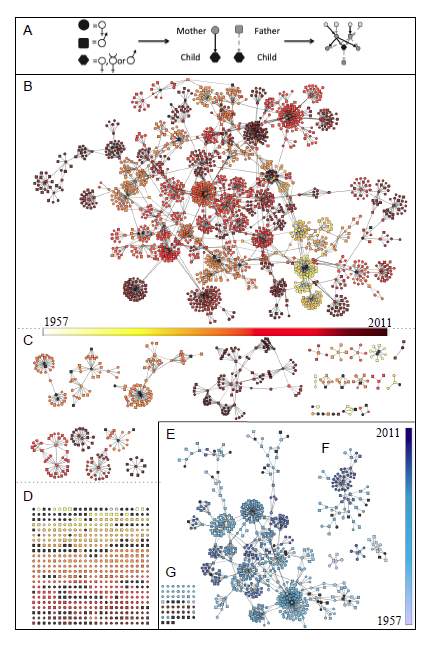


**Figure S1.** **Genealogies represented as kinship directed acyclic graphs (kDAGs).** Each individual is represented as a node and parent nodes are connected to offspring nodes by directed edges (A), for the North American (B–D) and Australasian (E–F) meerkat populations. Both graphs collapse to one major cluster (B, E), many smaller unconnected sub-graphs (C, F), and a set of unconnected nodes (D, G). Variation in birth dates is indicated by color gradients. Individuals with inaccurate (to month) or unknown dates of birth are shown as black nodes.


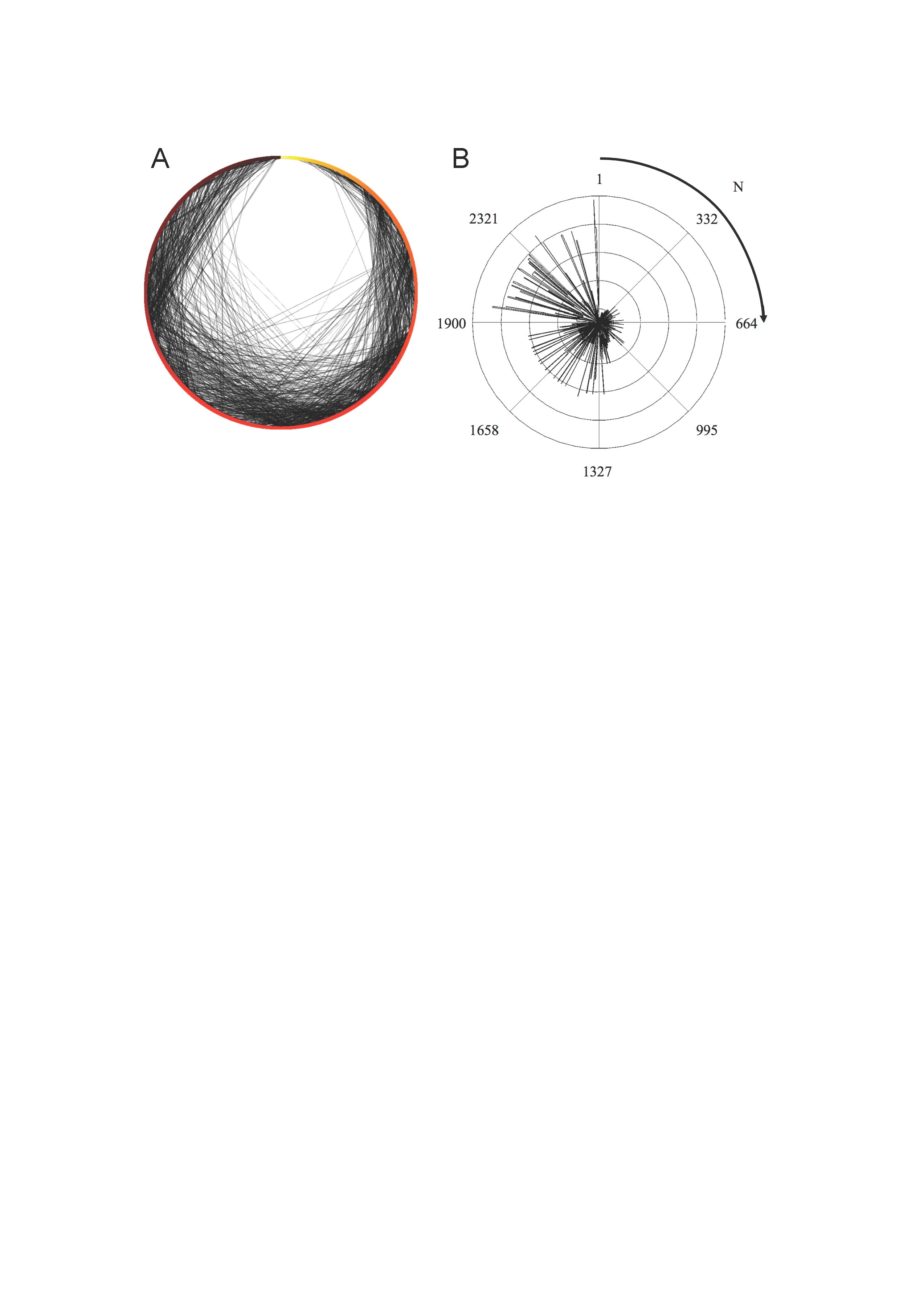


**Figure S2.** **The growth in network complexity within the kDAG showing edge effects and node connectedness.** By sorting the kDAG radially by order of entry into the population (date of entry indicated by color in A; order of entry (N) indicated by radial position in A and B), change in graph connectedness is indicated by the increasing density of directed edges over time (A, grey edges) and the closeness centrality of nodes (B). The closeness centrality of a node is a measure of the mean path length required to reach all nodes in a graph from that node (*16*). Unlike other centrality measures, closeness centrality incorporates information from all paths between nodes, not just the shortest mean path. Higher closeness centrality indicates higher connectedness, with a baseline closeness centrality of zero for an unconnected node.


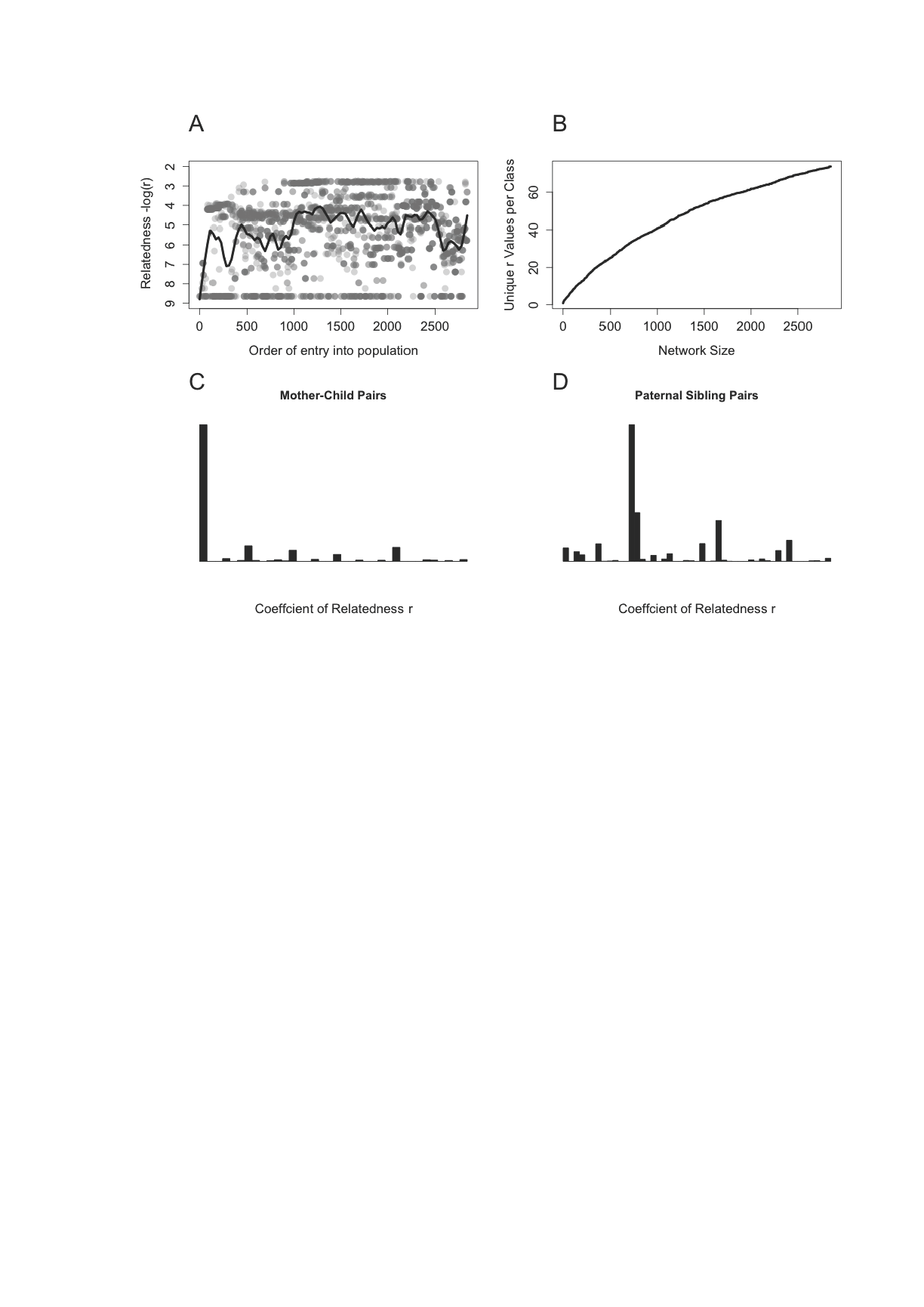


**Figure S3.** **Effects of increasing network complexity on calculated coefficients of relatedness, *r*, in the meerkat population.** (A) The mean coefficient of relatedness of focal individuals to the rest of the population is dependent on the order in which they enter the genealogy (either through birth or immigration) and is driven by changes in network connectedness and complex edge effects. (B) Individuals become increasingly connected by multiple relatedness paths as the diameter of the kinship network grows, increasing the number of unique values of *r* within each kinship category. (C) For instance, *r* between mother-child pairs increase from a single value (*r*=0.5) to 38 unique values as the kDAG increases in size. (D) A similar increase in unique *r* values occurs between paternal sibling pairs. Traditional kinship categories do not capture this variation in relatedness coefficients within the pedigree.


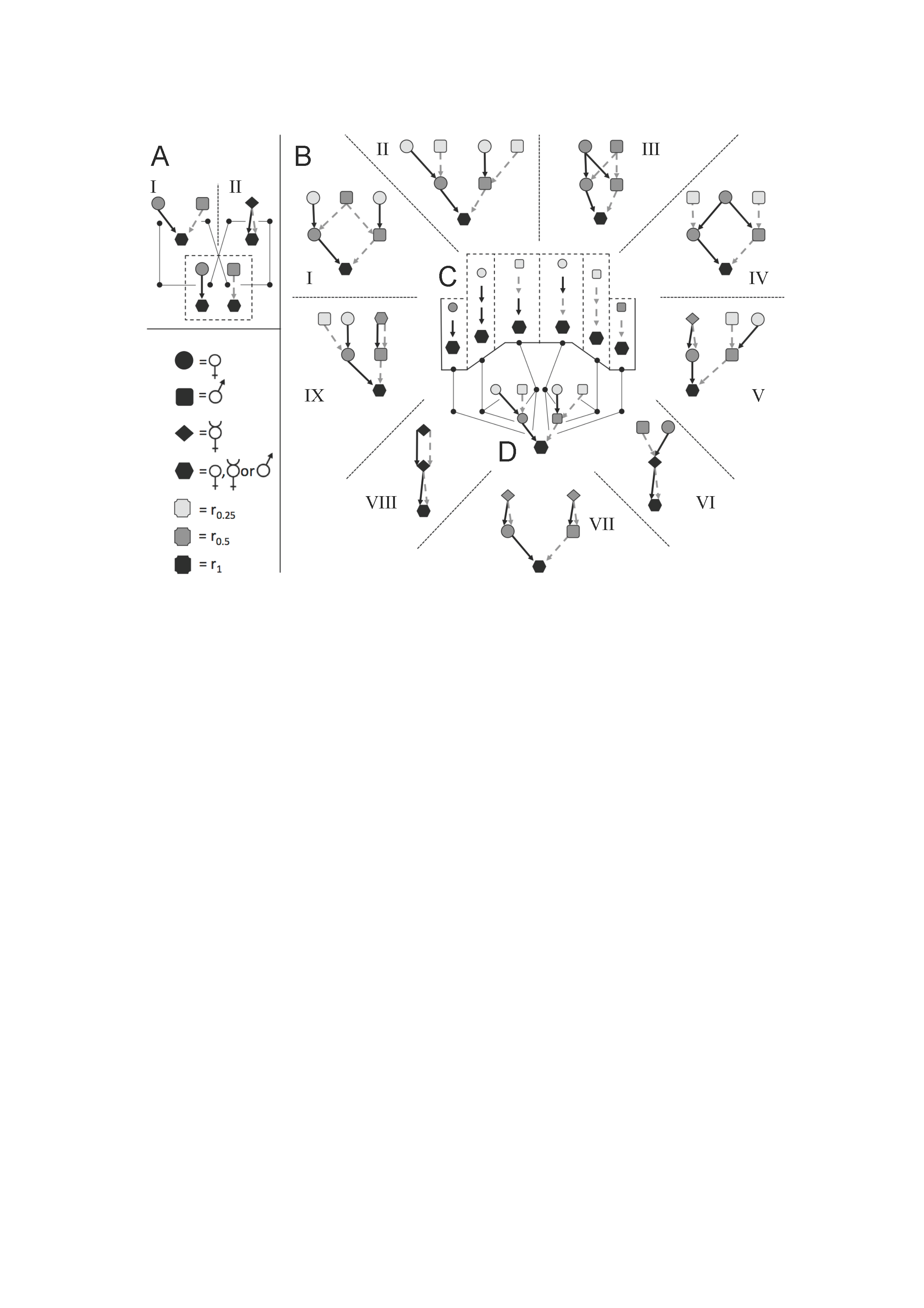


**Figure S4.** **The existence of a universal set of ascendant paths in diverse genealogies.** (A) For any strictly 2-sex species, a genealogy of first-degree ascendants has only two possible states: an individual has either one mother and one father (A I), or one selfing hermaphroditic parent (A II). These two genealogies contain different numbers of individuals, related by different coefficients of relatedness, but they can be represented by identical set of “paths” linking parents of each sex to their offspring. In both cases a child has exactly one female parent and one male parent (as indicated within dashed-line box). The number of possible genealogies is therefore larger than the number of possible path combinations. (B) This relationship holds at higher degrees of relatedness. At two degrees of relatedness, all 9 possible ascendant genealogies (B; I-IX) possess a fixed set of exactly one each of the 6 paths shown in (C). Each child has exactly one female parent, one male parent, one maternal and one paternal grandfather, and one maternal and one paternal grandmother, even if e.g., their maternal grandfather and their paternal grandfather is the same individual (as in B I). In this way, all genealogies of any given diameter have a single invariant set of ascendant paths, independent of their actual shape. (D) This invariant set of paths can be represented as a directed acyclic graph in which each node represents a class of relative (e.g., maternal grandfather, paternal grandfather, etc.), rather than a distinct individual. An individual may occupy more than one node (as e.g., in B I, where the same individual is both maternal grandfather and paternal grandfather), but a node may not contain more than one individual in an ascendant lineage.


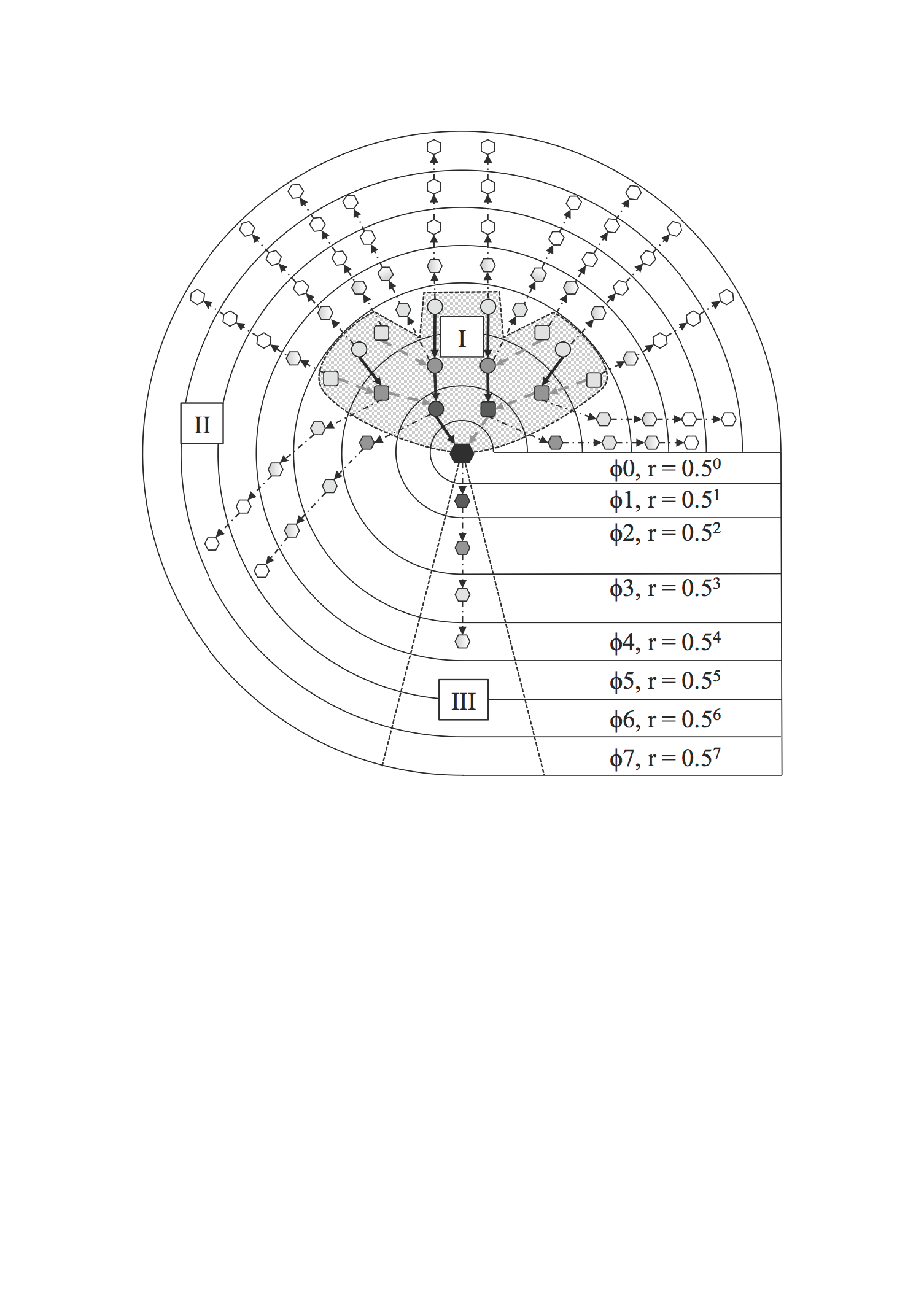


**Figure S5. The use of SIGs in model testing.** A 3-degree ascendant, 4-degree descendent SIG is shown with ascendant (I), descendant (III) and lateral (II) kin path sets identified as described in Fig. 1. SIGs were constructed for all focal individuals (represented by the black node) who have a complete set of documented third-degree ascendant relatives (I), in which all their known relatives are represented. For each focal individual, the numbers of local and global kin (local kin and global kin variables in Table 1) in each ascendant (I) and lateral (II) kin class were counted each month. We estimated how well the reproductive success of focal individuals (descendent relatives in path set III) was predicted by cohabitation of kin classes in path sets I and II. ϕ = degree of relatedness.


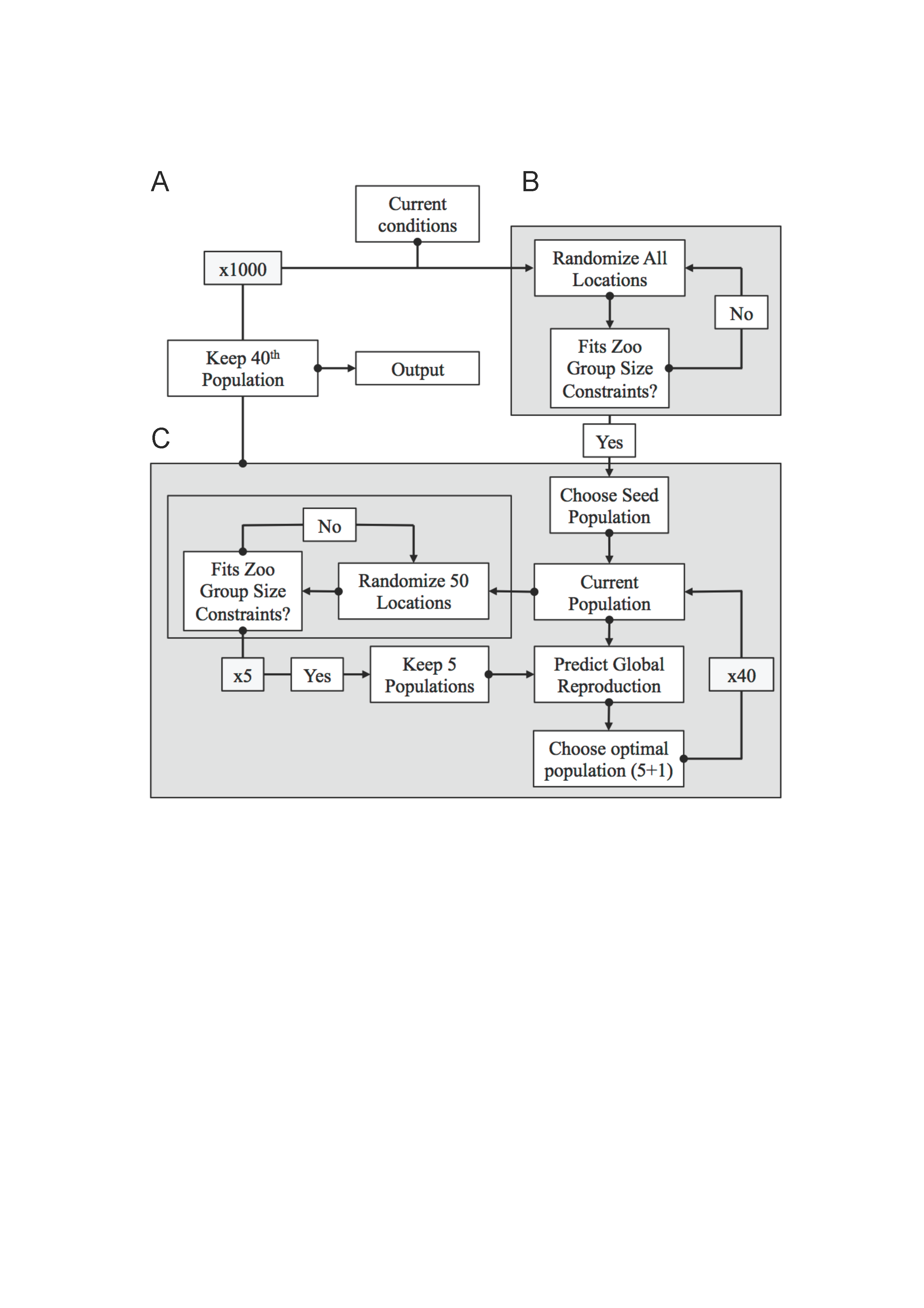


**Figure S6.** **Map of the genetic/packing algorithm used to optimize the social group structure.** (A) We generated 1000 seed populations that satisfied group size constraints for all zoos, with individuals randomly assigned to locations. (B) A local optimization algorithm was applied to each seed population. This algorithm generated “mutated” populations that randomly reassigned new locations to groups of 50 individuals from the seed population, rejecting populations that did not satisfy group size constraints, to generate five new populations. We forecast the overall reproductive success for these five “mutated” populations and the original population, and selected the global optimum of all six populations. (C) The globally optimal population was then used to start the next cycle of mutation-selection, and the total process repeated 40 times.

**Table S1.** Group size reduction cutoffs used in the combined genetic/ packing algorithm.

| Current group size (# animals) | Acceptable group size reduction |
| --- | --- |
| 1-5 | 0 |
| 6-8 | 1 |
| 9-12 | 3 |
| 13-15 | 4 |
| 16-19 | 5 |
| 20-25 | 8 |
| 25+ | Any reduction down to current holdings |
