# Supplementary material for: Reproductive success is predicted by social dynamics and kinship in managed animal populations
Source: F1000Res. 2016 May 11;5:870. [Version 1] doi: 10.12688/f1000research.8713.1 (PMC5155502; doi:10.12688/f1000research.8713.1)
Supplement: Peer review history

# Peer review: Referee response for version 1

**DOI:** 10.5256/f1000research.9375.r18031

**Published:** 2016-12-09

**Authors:** Alan H. Krakauer

**Affiliations:** 1 Department of Evolution and Ecology (EVE), University of California, Davis, Davis, CA, USA

The strength of this manuscript lies in its analytical methods. Treatment of data, use of a network framework for highlighting the role of different types of relatives, and the statistical models, are generally well described. I found it impressive that the models performed as well as they did on the independent dataset (models developed using North American records were tested on data from the Australian populations). The approaches described here might be useful beyond inbred tightly-managed captive conservation settings, and could potentially help reveal the importance of multiple types of genealogical relationships in complex societies.

 Two possible issues with appropriateness of the data: First, how parentage, and paternity in particular, is determined is not described in the text. Speaking with a zoo director about this, I learned that this is usually, but not always clear from behavior in the colony. Additionally, the text does not state how castrated individuals were treated (meerkat colony management occasionally involves neutering males to reduce aggression). The latter would obviously be extremely important for understanding direct reproductive success. The issue of whether human-sterilized individuals should be lumped together with “naturally” reproductively suppressed individuals is interesting in itself but not discussed in the manuscript. My guess is that neither of these issues change the data sufficiently to alter the outcome of the analyses, but it would be nice to know more.

 It does not appear that the analysis took into account the identity of the zoo. It seems plausible to me that zoos could differ in the quality of care and housing in ways that are confounded with their decisions about meerkat group management. Since I’m not sure this paper makes specific management recommendations and seems to be more proof-of-concept of the analytical methods, I don’t necessarily see this as too damning.

 I don’t think the distinction between “game theory” and “kin selection theory” is necessarily accurate, nor particularly useful for the message of the paper. For example, see the following for a recent paper which uses a game theoretic approach to ask behavioral questions in an inclusive fitness context: Akçay &Van Cleve, 2016 ^1^.

I have read this submission. I believe that I have an appropriate level of expertise to confirm that it is of an acceptable scientific standard.

## References

[1] AkçayEVan CleveJ: There is no fitness but fitness, and the lineage is its bearer. *Philos Trans R Soc Lond B Biol Sci*.2016;371(1687) : 10.1098/rstb.2015.0085 20150085 10.1098/rstb.2015.0085 26729925PMC4760187

# Peer review: Referee response for version 1

**DOI:** 10.5256/f1000research.9375.r17367

**Published:** 2016-11-02

**Authors:** Tim Clutton-Brock, Josephine Pemberton

**Affiliations:** 1 Large Animal Research Group, Department of Zoology, University of Cambridge, Cambridge , UK; 2 Institute of Evolutionary Biology, School of Biological Sciences, University of Edinburgh, Edinburgh, UK

All fine – but the conclusions are incorporated in a way that makes them difficult for readers to assimilate. Both the Abstract and the Results need to explain how kin and group dynamics affect breeding success (i.e. which components of fitness are affected, in which direction and to what extent). The authors also need to be clearer about how and why they believe their results affect game and inclusive theory interactions; how they reconcile predictions based on these two approaches; and why they believe that the results of studies of captive animals of this kind provide insight into the evolutionary processes operating in natural populations.

We have read this submission. We believe that we have an appropriate level of expertise to confirm that it is of an acceptable scientific standard.
